# Alginate-Based UV Sensor: A Simple and Inexpensive Tool for Educational Purposes

**DOI:** 10.1021/acs.jchemed.4c00291

**Published:** 2024-07-09

**Authors:** Kariluz Dávila-Díaz, Liz M. Díaz-Vázquez

**Affiliations:** University of Puerto Rico, Rio Piedras Campus, 17 Ave Universidad STE 1701, San Juan, Puerto Rico, 00925-2537

**Keywords:** General Public, Elementary/Middle School Science, Demonstration, Alginate-Based Sensors, Cross-Linking, Hands-on Learning, UV Radiation, Sensors, Applications of Chemistry

## Abstract

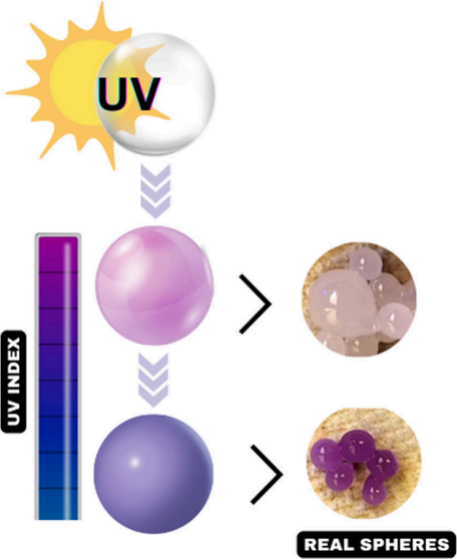

This document presents a simple yet highly effective demonstration for creating UV radiation sensors using alginate molecules. This demonstration can easily be aligned with the Next Generation Science Standards (NGSS) for classroom use. Moreover, the demonstration requires only a few easily obtainable materials, and the process involved is straightforward. When exposed to UV light or sunlight, the spheres’ color changes, offering a fascinating observation that is sure to capture the imagination of students of all ages. This encourages curiosity and inspires further exploration of the scientific world. It is easily understandable and suitable for people of all ages. This experiment represents a valuable addition to the scientific community’s educational tools, and its potential to inspire a new generation of scientists is truly limitless.

As the world changes and we face the depletion of the ozone layer, we face the consequence of increased exposure to the sun’s rays, including ultraviolet radiation. Understanding that radiation exposure can have severe implications for our health is crucial. For instance, extended exposure to UV rays can significantly increase the risk of skin cancer and may even lead to the development of cataracts. To avoid these harmful effects, it is imperative to take the necessary precautions to protect ourselves from radiation exposure and ensure our safety.^1^ Therefore, there is a growing demand for educational tools that teach the importance of measuring and understanding these rays.^2^ These tools must not only elucidate the science behind UV radiation but also engage learners in a meaningful exploration of its impacts. Astronauts that go to space must be aware if they are exposed to radiation. For example, the Artemis mission goal of NASA is to put astronauts on the moon.^3^ These astronauts need to be safe by knowing when they are exposed to radiation, for example, UV radiation. The need for radiation awareness is reflected here on Earth, where climate change is causing more frequent extreme heat waves and increased UV radiation levels compared to previous decades.^4^ While there are sensors capable of detecting radiation, there is a compelling need to create accessible versions that can be used by children and the general public.

Sensors^5^ are defined as “a device that can react to light, heat, pressure, etc. in order to make a machine, etc. do something or show something”. Using readily available and safe materials, we aim to explain UV radiation and foster a deeper understanding of its effects and who to monitor exposure to it. Through a detailed exposition of the sensor fabrication process involving the interaction of alginate molecules and calcium ions, this work is designed to spark curiosity and dialogue among students of all ages. The proposed demonstration aligns with current educational goals to enhance scientific literacy in both formal and informal settings, emphasizing the importance of innovative methods in teaching complex scientific concepts.

Commercial UV beads, available in a variety of colors, are readily accessible. These UV beads have photochromic pigments embedded into a polyethylene or polypropylene base, so they might be a good starting point to talk about radiation and protection. However, strategies that allow students to experience and feel that they are doing science with their hands but also attract them to pursue careers in STEM are necessary to increase the need for a workforce in these areas. Maltese and Tai^6^ (2010) reported that students from young ages who showed interest in science before middle school were more likely to complete a career in science. They also mentioned that teachers should focus on initiating and fostering engagement for young students to continue in science instead of just examining their knowledge. Kontra et al.^7^ concluded that physical, hands-on experiences will help students better understand science. Students lose interest in science compared to elementary and secondary school students.^8,9^

Sensors of photochromic alginate beads can easily be made using easy-to-find and safe-to-handle materials. It is important to note that commercially available beads are derived from petrochemicals, whereas alginate beads are not. Photochromic pigments can change color due to a reversible phototransformation of their structure and absorb in a different region of the electromagnetic spectrum.^10−13^ Potochromism has several applications. These applications include sunglasses lenses, data storage, toys, cosmetics, clothes, supramolecular chemistry, and solar energy storage.^14^ Several photochromic compounds include nitrones,^15^ fulgides,^16^ naphthopyrans,^17,18^ spiropyrans, spiro-oxazines,^19,20^ quinones,^21^ stilbenes,^22^ and azastilbenes.^23,24^ Many of these have been used in the fabrication of sensors. The fundamental principle of the use of photochromic pigments in the fabrication of sensors is described in Figure 1.

**Figure 1:**
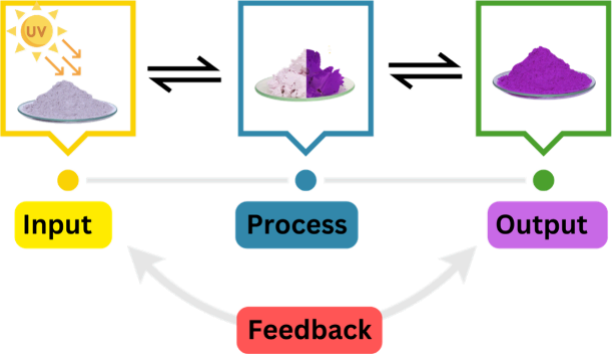
Schematic illustration of a photochromic pigment-based sensor. The “Input” shows photochromic pigment exposed to UV light, initiating the “Process” where a color change occurs due to UV exposure. The “Output” demonstrates the visible result of this process: the pigment’s color change. The “Feedback” loop indicates the reversible nature of the photochromic reaction, allowing the pigment to return to its original state in the absence of UV radiation, ready for subsequent cycles of detection.

Building upon foundational work with alginate spheres,^25−29^ our demonstration introduces an updated approach by integrating photochromic technology to generate UV-responsive beads. These beads provide a visual response to UV radiation, enabling students to understand phototransformation in a tangible way. This modification extends the pedagogical utility of alginate beads, linking chemical kinetics with environmental monitoring.

These beads were made using the spherification process. As a scientific technique, spherification involves creating spherical encapsulations of various substances, such as cells, compounds, or nanoparticles. This process takes advantage of the gelling properties of alginates in the presence of divalent cations, typically calcium. The key point about this is the production of small, jelly like spheres.

Alginate is a polysaccharide derived from brown seaweeds^30,31^ that starts to organize around divalent cations such as calcium, creating a gel-like membrane around a liquid center.^32^ The interaction between alginate strands and calcium has been described as the “egg-box” model^33^ (Figure 2), where the calcium ions interact with two alginate strands, cross-linking them. This process is crucial in facilitating the formation of the membrane. Alginate is used in science for a variety of applications, including spherification. Since it is nontoxic, nonantigenic, biocompatible, and biodegradable, alginate is generally regarded as safe (GRAS).^34^

**Figure 2:**
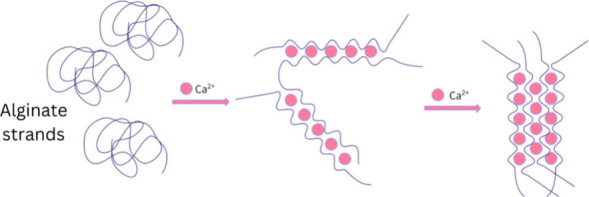
Egg-box model for the alginate spherification process. This model allows us to explain how the membrane is formed when exposed to calcium ions.

Alginate spherification/gelling has several uses and applications. Some of these usages and applications^35,36^ are microencapsulation,^37−42^ controlled release systems,^43−46^ sensors development,^47−50^ and food and flavor encapsulation,^51,52^ among others.

Radiation detection is a critical aspect of NASA missions, particularly when it comes to ensuring the safety of astronauts in space. Due to the absence of an atmosphere in space, astronauts are more exposed to space radiation, which can be harmful to their bodies. To overcome this challenge, NASA uses a range of tools, including sensors on spacecraft and suits, to monitor radiation levels and keep astronauts safe. Sensors are critical to the success and safety of space missions, allowing spacecraft to operate safely, collect scientific data, navigate accurately, and adapt to changing conditions in space. They play a vital role in ensuring the success of space exploration endeavors, and their importance cannot be overstated. Without sensors and other radiation detection tools, NASA missions would not be possible, and the safety of astronauts would be at risk. The innovative photochromic alginate beads are a valuable educational tool. These beads change color in UV light, similar to how materials can indicate the presence of radiation.

This demonstration can catalyze a discussion of reversible reactions and the importance of protecting oneself when exposed to sunlight. Talking to kids and demonstrating UV-sensors made with alginate and photochromic pigments has value in education and awareness by allowing them to recognize the risks of UV radiation^53^ in their daily lives and introducing them to science and technology interactively and engagingly by having hands-on learning, building confidence and creative expression, and problem-solving skills.

## LEARNING GOALS

The following table (Table 1) delineates a structured sequence of learning goals associated with the use of photochromic alginate beads as a teaching tool. These goals are designed to introduce and expand upon key scientific concepts, each aligned with specific Next Generation Science Standards (NGSS).^54^ From the fundamental properties of light in elementary classrooms to the complex interactions of molecular chemistry at the university level, this activity is crafted to support knowledge, stimulate inquiry, and foster interest in chemistry.

**Table 1:** Progressive Learning Goals for Photochromic Alginate Bead Activities Across Educational Levels

Educational Level	Concept	Activity Approach	NGSS standard
Elementary	Introduction to Light	Demonstrate how sunlight changes the color of beads; simple explanation of UV light.	2-PS1-1
	Basic Chemistry	Show mixing of alginate and calcium ions creates beads; discuss observable properties.	2-PS1-1
Middle School	UV Radiation and Protection	Explain UV light’s role and effects; introduce UV protection using beads.	5-PS1-3
	Particle Model of Matter	Discuss matter’s particle nature, using beads to model atoms and molecules too small to be seen.	5-PS1-1
	Chemical Reactions	Investigate how substances interact during bead formation to determine if a chemical reaction has occurred.	5-PS1-4, MS-PS1-2
High School	Photochromism	Explore molecular structure changes in beads upon UV exposure; discuss chemical properties and reactions.	HS-PS1-2
	Environmental Science	Discuss environmental impacts of UV radiation, linking to synthetic materials from natural resources	MS-PS1-3
Undergraduate	Polymer Chemistry	Examine the polymerization in bead formation; discuss alginate as a polymer and its properties.	(Advanced application)
	Spectroscopy	Analyze beads’ color change using spectroscopy; understand different spectrum regions’ absorption.	(Advanced application)
Adult/Informal Education	Science Communication	Engage the public in science discussions using beads to explain climate change, UV monitoring, and scientific literacy.	(Community engagement)

## MATERIALS

All the materials used for this demonstration were acquired through Amazon.com. sodium alginate (100% food grade), calcium lactate (food grade), and the photochromic pigment purple were used as received. The UV-photochromic pigment comes in several colors. Any color should give similar results. The white to purple was selected for its high contrast and visibility. A UV source (Mini UV LED keychain flashlight (395 nm UV) was used), clear plastic reusable zip bags (1.5″ x 2″), plastic mini transfer pipettes (0.2 mL), disposable coffee teaspoon-stirrer, and graduated medicine cups were also acquired through Amazon.com and used as received. Room-temperature bottled water and paper towel rolls were acquired at a local supermarket to prepare the solutions. A list of materials has been included as Supporting Information.

## HAZARDS AND WASTE DISPOSAL

Sodium alginate, calcium lactate, and photochromic pigment are not considered hazardous substances. However, wearing protective goggles, safety gloves, and a protective coat is necessary while handling these materials. It is important to emphasize that the reagents or the products from this demonstration should not be consumed even if the used materials are classified as “food grade”. When using UV flashlights or sunlight to cause color change, it is important to avoid looking directly into the light sources.

The safety data sheet for alginate and calcium lactate can be accessed using the following links sodium alginate^55^ and calcium lactate.^56^

## PROCEDURE

It is important to follow the recommendations in the Safety Guidelines for Chemical Demonstrations of the ACS before starting the demonstration.^57^ A document with instructions for the demonstration has been included in the Supporting Information.

**Figure 3:**
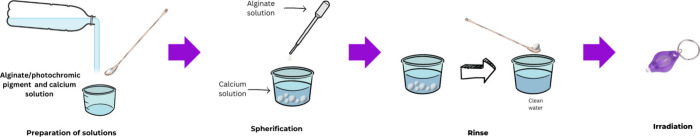
Simplified outline of the demonstration procedure, starting with the preparation of the solutions, spherification, rinse, and at last the irradiation of the beads with UV-light.

To prepare the necessary solutions for spherification (sodium alginate and calcium lactate solutions), we developed a modified version of Sen’s^58^ procedure. We prepared the alginate mix by adding and mixing the photochromic pigment with the sodium alginate solid. The weight percentage of the photochromic pigment in the alginate mix was approximately 10%. A batch was prepared from which it was divided for the different demonstrations. It was very important to homogenize the solids, so the pigment is throughout the mixture. Before preparing the alginate solution, as both solids were initially white, we were able to check the homogeneity of the mixture by irradiating it with UV. The photochromic pigment will change color and the alginate will stay white, easily seeing the distribution in the mixture. A simplified outline of the demonstration procedure is shown in Figure 3.

Participants prepared both solutions in a graduated medicine cup at around 1% weight concentration. The alginate solution was prepared with 0.05 g of the alginate mix in 5 mL of water and the calcium lactate solution with 0.1 g in 10 mL of water. It is recommended that the person to lead the demonstration measure the recommended amount before the demonstration so that he/she can guide the participants according to the available instruments to be used. This alginate mix was added to one of the medicine cups, and room-temperature water was added. The alginate solution should be opaque. The calcium solution was made using calcium lactate because it is a dietary supplement but can also be made with calcium chloride or any soluble calcium salt. The calcium lactate took a couple of seconds to dissolve, but the sodium alginate took a few minutes and required more assistance to dissolve. A separate spoon-stirrer was used for each solution to minimize cross-contamination and ensure that the demonstration worked properly.

**Figure 4:**
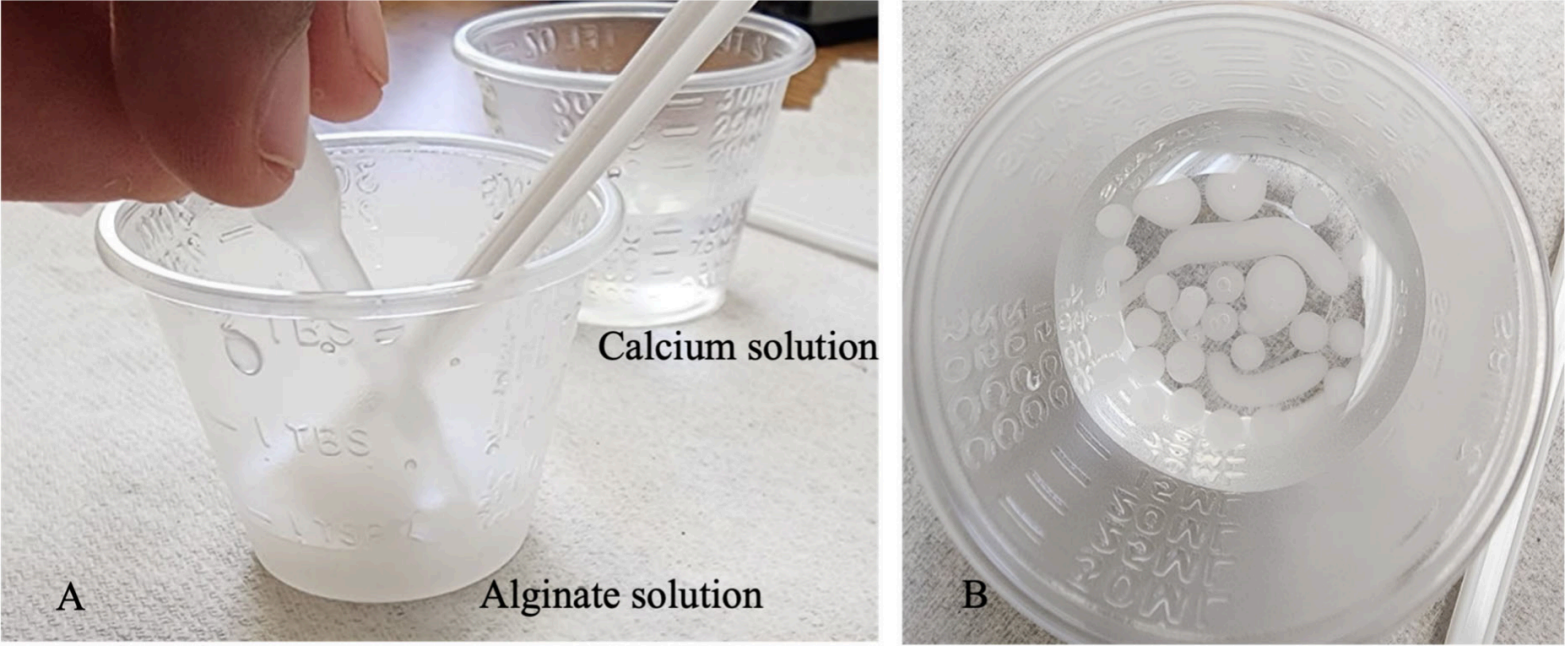
(A) A dropper or transfer pipet was used to form the beads. The spoon-stirrer was also used to drop bigger beads. (B) Top view of the beads formed when the drops of alginate solution were added to the calcium solution. A string of alginate can be seen. This occurs when the alginate solution is added too quickly with the dropper.

The alginate solution was added drop-by-drop to the calcium solution. The tip of the dropper cannot be introduced inside the solution. Adjusting the drop size allows for control over the size of the sphere/bead, resulting in precise and consistent outcomes. We used a 0.2 mL transfer pipet, forming drops of approximately 3–5 mm in diameter. The drops can be added using other droppers or spoons, which will vary the size of the final sphere/bead size. The spheres/beads were left in the calcium solution for at least 30 s (Figure 4B). This process will help the alginate create a gel-like membrane around its liquid center. The longer the spheres stay in the calcium solution, the firmer their surface becomes.

To rinse the alginate beads, participants measured 10 to 15 mL of room-temperature water in a third graduated medicine cup. Participants used a spoon to transfer the beads from the water to a third cup. This allowed the spherification process to stop and remove any calcium lactate from the surface. Once the spheres were rinsed, they were removed from the water and put on a paper towel. Once the spheres were rinsed, the spheres/beads could be manipulated.

**Figure 5:**
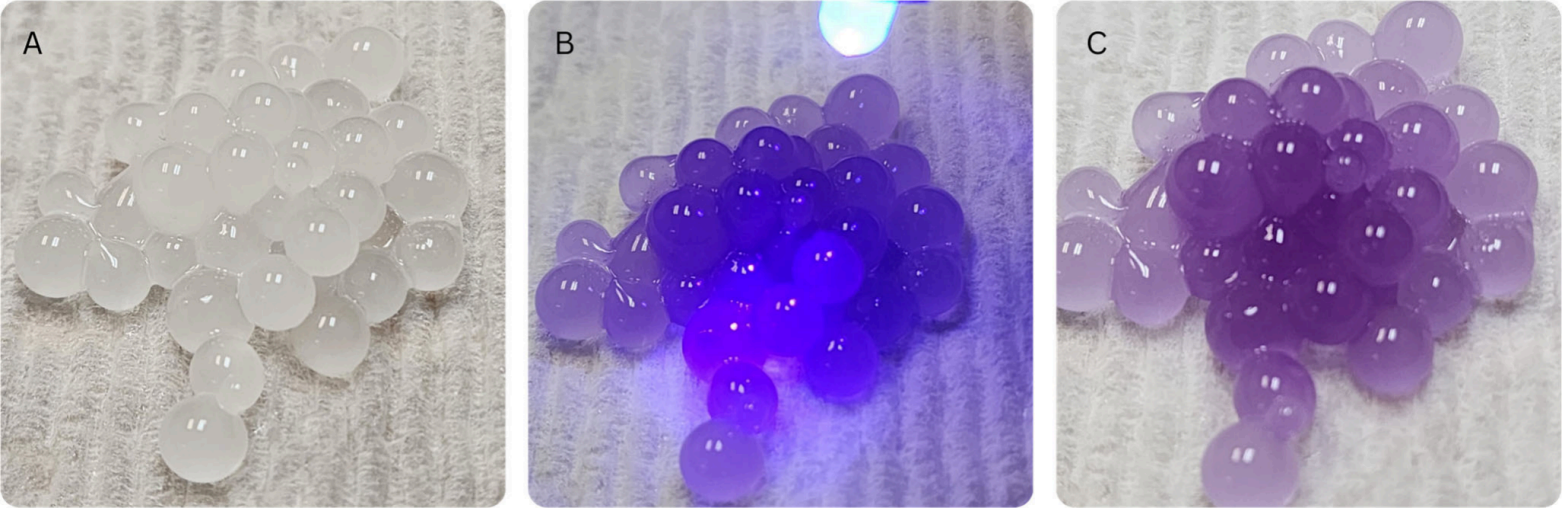
(A) Rinsed alginate beads under room light. They look white as the alginate solution. (B) The alginate beads are exposed to UV light using a UV light flashlight. Another source that can be used is sunlight. (C) Alginate beads after a quick exposure to UV light. It can be seen that the beads changed color to purple since the photochromic pigment was white changing to purple. The color of the beads after exposing them to UV depends on the photochromic pigment used.

The spheres will not change color unless they are exposed to UV light (Figure 5A). UV light can come from a UV flashlight or the sunlight. The color change that will be observed will depend on the pigment used. For our demonstration, the photochromic pigment used was white in its colorless form and purple in its colored form. When exposed to UV light or sunlight, the spheres changed color immediately (Figure 5B and 5C). The longer the beads were exposed to UV radiation the deeper the color. This demonstration has been used in rooms where there are no windows and where an entire wall is made of glass. In both scenarios, the color has not changed significantly without exposure to ultraviolet light. When the UV light is stronger in the room a faint color can be noticed however it is almost imperceptible. The spheres respond to ultraviolet light whether they are in or out of solution or water. Temperature is known to affect the rate of color change of photochromic pigments. This has been done with commercial UV beads.^59,60^ It opens to make a variation of the demonstration by applying different temperatures and observing the effect. It is also known that different photochromic pigments have different rates of change so this variation can be explored with different pigments and different temperatures. Several variants of experiments can be done with the alginate beads, for example seeing if sunscreen or any colored filter can block UV rays.

To ensure the beads remained fresh and usable, they were stored in a zip-top bag. This method is highly effective in maintaining the quality of the beads for weeks, providing ample time to utilize them as required. A shelf life study of the spheres was not performed, but after one month, the spheres were still formed when stored in the bags. A change in shape was seen, but not in the response to UV radiation. We did not notice any mold formation.

Also, an advice for practitioners is to become familiar with the process before implementing it. Depending on available equipment and materials, they would like to make adjustments to the procedure. In addition, doing it in advance and familiarizing with the process allows foreseeing difficulties that may be encountered by the population with whom the demonstration will be performed.

## DISCUSSION

The use of attractive and colorful demonstrations^61−65^ in chemistry offers numerous advantages, commonly in educational settings. The advantages of this include enhanced engagement, concept visualization, and real-world application. Enhanced Engagement: capture students’ attention and make the learning experience more engaging. Visual appeal can stimulate interest and curiosity, fostering a positive learning environment. Concept Visualization: Abstract concepts in chemistry can be challenging to grasp. These demonstrations assist students in visualizing concepts by providing a tangible representation of chemical reactions and phenomena. Real-world Application: often simulates real-world applications of chemical principles. This practical connection allows students to understand the relevance of chemistry in everyday life and various industries.

This demonstration has been used with adults, children, and adolescents from 12 years of age and older. The feedback from the adults who participated in the demonstration was very positive. They all wanted to use it in their activities, whether in informal education, camps, or the classroom. As for the students, they were attracted by the demonstration, generating questions about UV exposure, alginate polymerization, and the use of sensors in various settings.

Through this demonstration, students were able to comprehend the difficulties posed by space radiation, appreciate the value of sensors in space missions, and do experiments with materials that react to changes in their surroundings.

The demonstration was an outstanding educational tool for the students. It was both engaging and informative, leaving a lasting impression on them. The experiment not only sparked their curiosity but also gave them a chance to play the role of scientists. It allowed them to explore various aspects of the experiment, including exposure time, shapes, and tactile feedback. The demonstration played a crucial role in stimulating their interest in the subject and encouraging them to delve deeper into the scientific world.

## CONCLUSION

This paper describes a straightforward method for creating UV radiation sensors utilizing calcium ion-conjugated alginate molecules. This experiment can be a teaching tool for people of all ages because it uses simple procedures with readily found, safe materials. It aims to promote productive discussion on photochromism, spherification, polymerization, UV light protection, and sensors.

In summary, this demonstration can serve as a source of inspiration for upcoming scientists and is an excellent addition to the educational materials available for scientific educators.

## Supporting Information Available

Color changing alginate beads teacher (PDF)
Color changing alginate beads teacher, Spanish (PDF)
Color changing alginate beads brochure (PDF)
Color changing alginate beads brochure, Spanish (PDF)
