# Supplementary material for: Alginate-Based UV Sensor: A Simple and Inexpensive Tool for Educational Purposes
Source: J Chem Educ. 2024 Jul 9;101(8):3596–602. doi: 10.1021/acs.jchemed.4c00291 (PMC11328127; doi:10.1021/acs.jchemed.4c00291)

Supporting Information

Alginate-based UV Sensor: A Simple and Inexpensive Tool for Educational Purposes

Kariluz Dávila-Díaz*, Liz M. Díaz-Vázquez
University of Puerto Rico, Rio Piedras Campus
17 Ave Universidad STE 1701
San Juan PR 00925-2537

*kariluz.davila@upr.edu

Color changing Alginate Beads as Sensor for UV-Radiation: Educational Tool

Color changing Alginate beads as Sensor for UV-Radiation: Educational Tool

Information

As the world changes and we face the depletion of the ozone layer, we face the consequence of increased exposure to the sun's rays, including ultraviolet radiation. Understanding that radiation exposure can have severe implications for human health is crucial. For instance, extended exposure to UV rays can significantly increase the risk of skin cancer and may even lead to the development of cataracts. To avoid these harmful effects, we must take the necessary precautions to protect ourselves from radiation exposure and ensure our safety. Therefore there is a growing demand for educational tools that help teach the importance of knowing when exposed to these rays. These tools must elucidate the science behind UV radiation and engage learners in a meaningful exploration of its impacts. Astronauts that go to space must be aware if they are exposed to radiation. For example, the mission goal of NASA's Artemis mission is to put astronauts on the moon. These astronauts need to be safe by knowing when they are exposed to radiation, such as UV radiation. This necessity for radiation awareness is mirrored here on Earth, where climate change leads to more frequent extreme heat waves and increased radiation levels compared to previous decades. While sensors capable of detecting radiation exist, there is a compelling need to create accessible versions that can be used by children and the general public.

Sensors of photochromic alginate beads can easily be made using easy-to-find and safe-to-handle materials. **Photochromic pigments** can change color due to a reversible phototransformation of their structure and absorb in a different region of the radiomagnetic spectrum. Photochromism has several applications. These applications include sunglasses lenses, data storage, toys, cosmetics, clothes, supramolecular chemistry, and solar energy storage. Many of these have been used in the fabrication of sensors. The fundamental principle of using photochromic pigments in fabricating sensors is described in Figure S1.

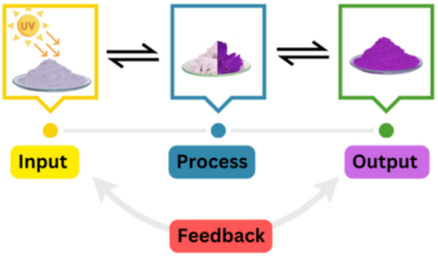

Figure S1. Schematic illustration of a photochromic pigment-based sensor. The 'Input' shows photochromic pigment exposed to UV light, initiating the 'Process' where a color change occurs due to UV exposure. The 'Output' demonstrates the visible result of this process: the pigment's color change. The 'Feedback' loop indicates the reversible nature of the photochromic reaction, allowing the pigment to return to its original state in the absence of UV radiation, ready for subsequent cycles of detection.

Alginate is a polysaccharide derived from brown seaweeds that starts to organize around divalent cations such as calcium, creating a gel-like membrane around a liquid center. The interaction between alginate strands and calcium has been described as the “egg-box” model (Figure S2), where the calcium ions interact with two alginate strands, cross-linking them. This polymerization process is crucial in facilitating the formation of a membrane, as it involves the exposure of the alginate solution to calcium ions.

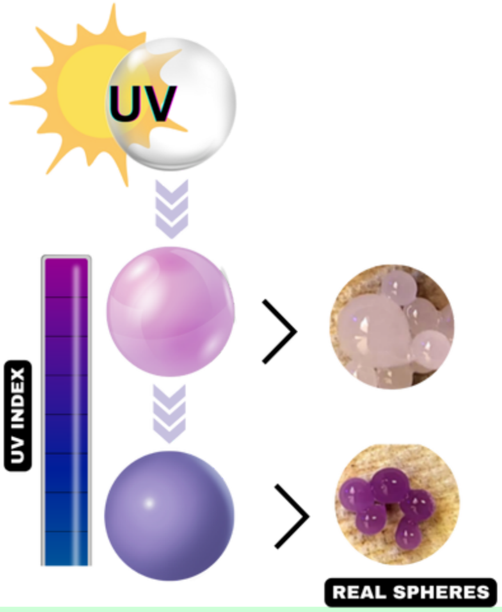

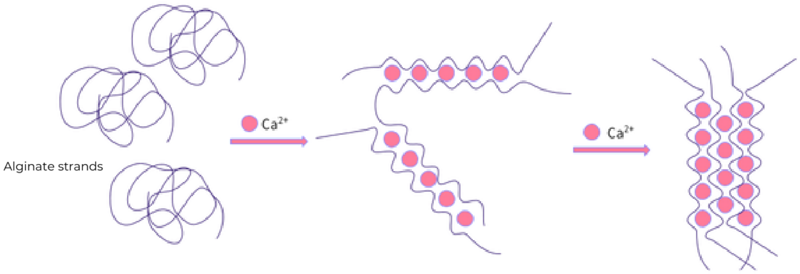

Figure S2. Egg-box model for the alginate spherification process. The blue lines represent alginate strands.

Some of the usages and applications for the alginate are controlled drug delivery, tissue engineering and cell encapsulation, flavor encapsulation within food science, microencapsulation of microorganisms, immobilization of enzymes and biocatalysis, artificial organs and cellular therapies, and Biosensors, among others.

These photochromic alginate beads can be an educational tool since they change color in UV light, simulating how materials can indicate the presence of radiation—similar to how sensors warn of high radiation levels in space. These beads respond to UV light just as sensors on spacecraft and suits will monitor various radiation levels to ensure astronauts' safety.

Color changing Alginate beads as Sensor for UV-Radiation: Educational Tool

Materials

- **Sodium alginate** (100% food grade)
  - Sodium alginate is derived from brown algae and is widely used in molecular gastronomy, so it is very easy to obtain. Sodium alginate is classified as "generally considered safe".
- **Calcium salt** (food grade)
  - Calcium solution can be prepared using any soluble calcium salt such as calcium lactate, calcium chloride, or calcium sulfate. Calcium lactate is commonly used to supplement people's calcium intake.
- **Photochromic pigment**
  - Any color of photochromic pigment can be used. It does not take much to see a color change. At least 10% photochromic pigment in the alginate mixture is enough to see a color change once the sphere is prepared.
- **UV light (395nm UV)**
  - Any source of UV light is enough to see a color change. If is not available a UV lamp or flashlight, direct exposure to the sun would be enough. A Mini UV LED keychain flashlight is recommended.
- **Eye dropper bottles or transfer pipette**
  - Droppers or transfer pipettes are used to add by drop the alginate solution into the calcium solution. If droppers or transfer pipettes are not available, adding the solution using the spoon is also an option. Beads using the spoon will be bigger, and more time spent in the calcium solution is recommended.
- **Stirrer/spoon**
  - The spoon/stirrer prepares the alginate and calcium solutions. They also remove the beads from the calcium solution and rinse water. Any spoon or stirrer can be used. We recommend using a small disposable coffee/tea spoon/stirrer to fit the cups better.
- **Graduated medicine cups**
  - The graduate medicine cups have two functions. The first function is to measure the water volume to be used to prepare the solutions or the rinse water. The other function is to prepare the solutions in them. These cups are small, easy to find, and clean.
- **Paper towel**
  - A paper towel will help clean the area, and the beads will be put on once they are out of the water.
- **Water**
  - Water bottles are recommended because they are easy to carry and store.
- **Clear plastic reusable zip bags** (1.5" x 2") (optional)
  - The zip bags serve to store the beads and extend their life. The alginate beads are made with water and, if exposed to air, can dehydrate and shrink. These zip bags can also be used to store the alginate mix (alginate + photochromic pigment) or the calcium salt (for example, the calcium lactate) for easy transport.

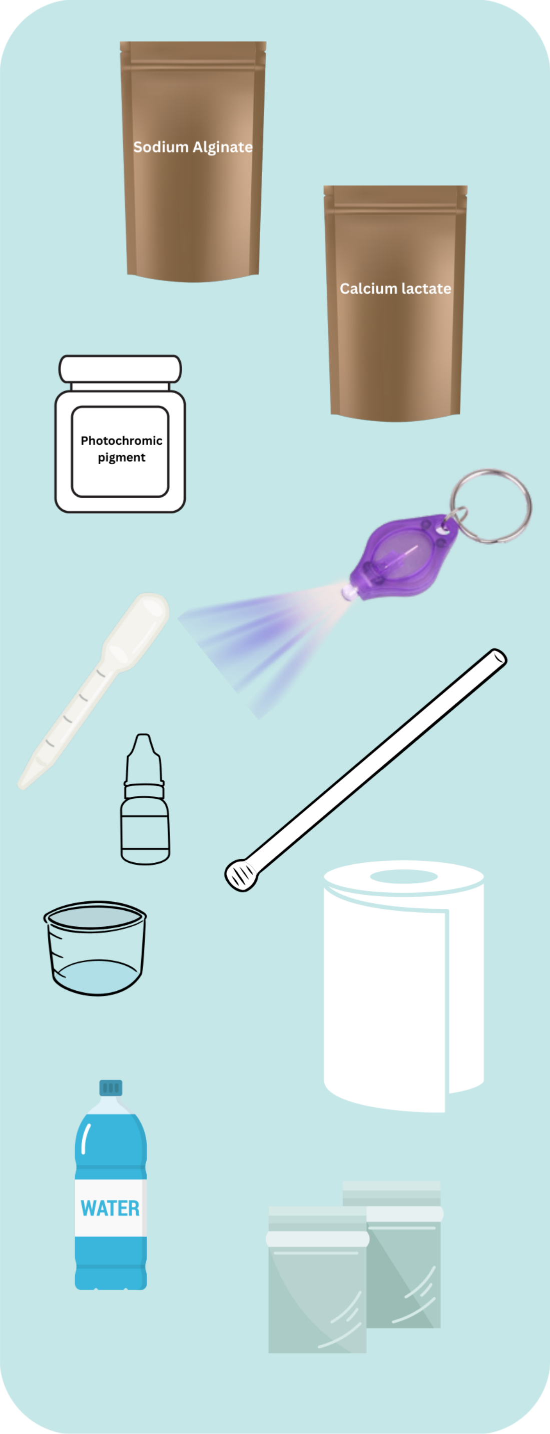

Proper precautions must be used when handling the materials. Always dispose of them safely, following local/national regulations. Consuming the spheres formed is not recommended.

Color changing Alginate beads as Sensor for UV-Radiation: Educational Tool

Procedure

Preparation

Alginate solution

- Alginate powder + photochromic pigment mix (10%)
- 5 mL water
- Measuring cup
- Stirrer-spoon

1. Measure 5 mL of water with one of the cups.
2. Add the tip of the spatula-stirrer of the alginate mix (~ 0.05 g)
3. Stir and mix until well blended. Use the back of the spoon to help dissolve any solids.
4. Set aside.

Calcium solution

- Calcium lactate
- 10 mL water
- Measuring cup
- Stirrer-spoon

1. Measure 10 mL of water with a clean measuring cup.
2. Add 2/3 of the spatula-stirrer (~ 0.1 g) of the calcium lactate to the water and mix using a new stirrer.
3. Mix until completely dissolved.
4. Set aside.

Spherification

- Calcium solution
- Alginate solution
- 10 - 15 mL of water
- Measuring cup
- Transfer pipette or spoon-stirrer

1. Add the alginate solution to the calcium solution drop by drop. Allow one drop to form before adding another. Do not introduce the tip of the dropper inside the solution.
2. Leave the spheres in the solution for at least 30 seconds.
3. Rinse the spheres. To rinse the spheres, remove them from the calcium solution with the spoon-stirrer and add them to fresh water.
4. Leave the spheres in the water for at least 30 s; then, put them in a clean cup or paper towel.
5. Let's see if they change color when applying UV light.

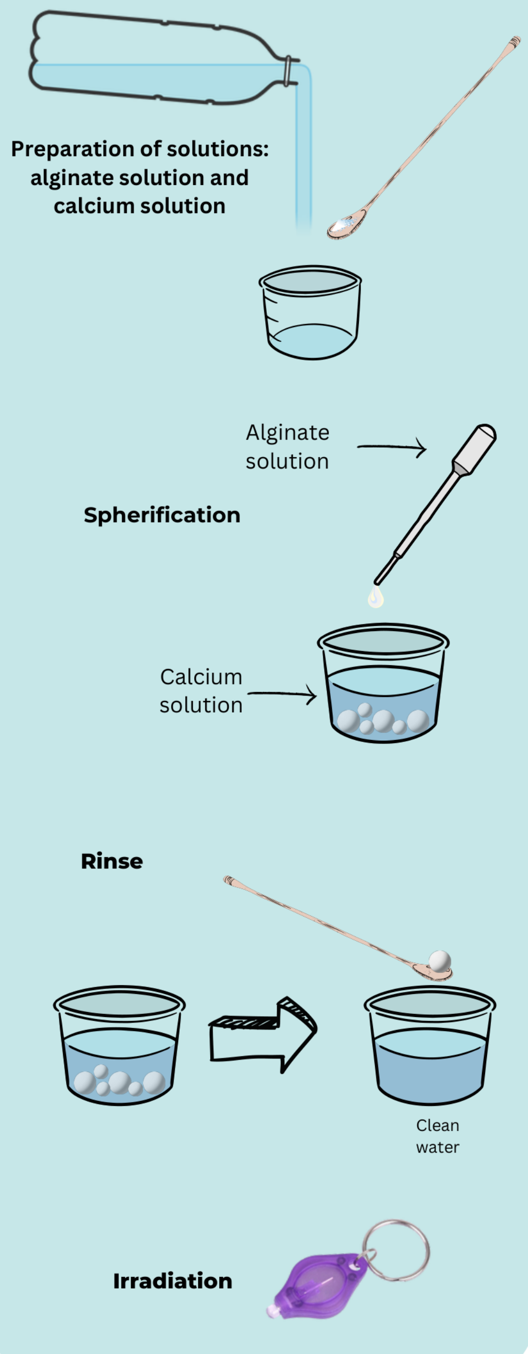

Color changing Alginate beads as Sensor for UV-Radiation: Educational Tool

Information

Progressive Learning Goals for Photochromic Alginate Bead Activities Across Educational Levels

Educational Level	Concept	Activity Approach	NGSS standard
**Elementary**	Introduction to light	Demonstrate how sunlight changes the color of beads; simple explanation of UV light.	2-PS1-1
	Basic Chemistry	Show mixing of alginate and calcium ions creates beads; discuss observable properties.	2-PS1-1
**Middle School**	UV Radiation and Protection	Explain UV light's role and effects; introduce UV protection using beads.	5-PS1-3
	Particle Model of Matter	Discuss matter's particle nature, using beads to model atoms and molecules too small to be seen.	5-PS1-1
	Chemical Reactions	Investigate how substances interact during bead formation to determine if a chemical reaction has occurred.	5-PS1-4, MS-PS1-2
**High School**	Photochromism	Explore molecular structure changes in beads upon UV exposure; discuss chemical properties and reactions.	HS-PS1-2
	Environmental Science	Discuss environmental impacts of UV radiation, linking to synthetic materials from natural resources.	MS-PS1-3
**Undergraduate**	Polymer Chemistry	Examine the polymerization in bead formation; discuss alginate as a polymer and its properties.	(Advanced application)
	Spectroscopy	Analyze beads' color change using spectroscopy; understand different spectrum regions' absorption.	(Advanced application)
**Adult/Informal Education**	Science Communication	Engage the public in science discussions using beads to explain climate change, UV monitoring, and scientific literacy.	(Community engagement)

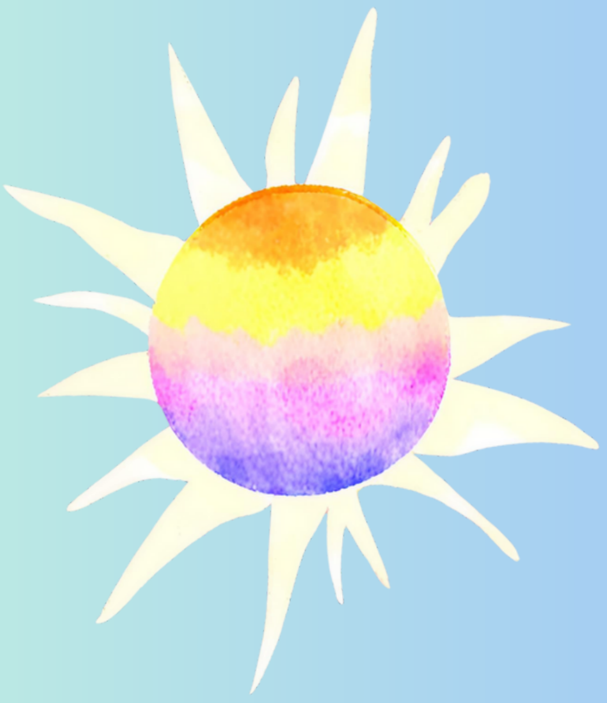

Supplement: Supplementary file 1 — ed4c00291_si_001.pdf [file ed4c00291_si_001.pdf]
