# Supplementary material for: Alginate-Based UV Sensor: A Simple and Inexpensive Tool for Educational Purposes
Source: J Chem Educ. 2024 Jul 9;101(8):3596–602. doi: 10.1021/acs.jchemed.4c00291 (PMC11328127; doi:10.1021/acs.jchemed.4c00291)

Supporting Information

Alginate-based UV Sensor: A Simple and Inexpensive Tool for Educational Purposes

Kariluz Dávila-Díaz*, Liz M. Díaz-Vázquez
University of Puerto Rico, Rio Piedras Campus
17 Ave Universidad STE 1701
San Juan PR 00925-2537

*kariluz.davila@upr.edu

Esferas de alginato que cambian de color como sensor de radiación UV: Herramienta educativa

Esferas de alginato que cambian de color como sensor de radiación UV: Herramienta educativa

Información

A medida que el mundo cambia y nos enfrentamos al deterioro de la capa de ozono, nos topamos con la consecuencia de una mayor exposición a los rayos del sol, incluida la radiación ultravioleta. Es crucial comprender que la exposición a la radiación puede tener graves consecuencias para la salud humana. Por ejemplo, una exposición prolongada a los rayos UV puede aumentar significativamente el riesgo de cáncer de piel e incluso puede provocar la aparición de cataratas. Para evitar estos efectos nocivos, es imprescindible tomar las precauciones necesarias para protegernos de la exposición a la radiación y garantizar nuestra seguridad. Por ello, existe una demanda creciente de herramientas educativas que ayuden a enseñar la importancia de saber cuándo nos exponemos a estos rayos. Estas herramientas no sólo deben dilucidar la ciencia que hay detrás de la radiación UV, sino también involucrar a los alumnos en una exploración significativa de sus impactos. Los astronautas que van al espacio deben saber si están expuestos a la radiación. Por ejemplo, el objetivo de la misión Artemis de la NASA es llevar astronautas a la Luna. Estos astronautas necesitan estar seguros sabiendo cuándo están expuestos a la radiación, por ejemplo, a la radiación UV. Esta necesidad de conocer la radiación se refleja aquí en la Tierra, donde el cambio climático está provocando olas de calor extremo más frecuentes y un aumento de los niveles de radiación en comparación con décadas anteriores. Aunque ya existen sensores capaces de detectar la radiación, hay una necesidad imperiosa de crear versiones accesibles que puedan utilizar los niños y el público en general.

Los sensores de esferas de alginato fotocrómico pueden fabricarse fácilmente con materiales fáciles de encontrar y seguros de manipular. Los pigmentos fotocrómicos pueden cambiar de color debido a una fototransformación reversible de su estructura y absorber en una región diferente del espectro radiomagnético. El fotocromismo tiene varias aplicaciones. Por ejemplo, lentes para gafas de sol, almacenamiento de datos, juguetes, cosméticos, ropa, química supramolecular y almacenamiento de energía solar. Muchas de ellas se han utilizado en la fabricación de sensores. El principio fundamental del uso de pigmentos fotocrómicos en la fabricación de sensores se describe en la Figura S1.

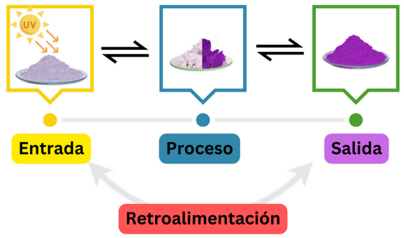

Figura S1. Ilustración esquemática de un sensor fotocrómico basado en pigmentos. La "Entrada" muestra el pigmento fotocrómico expuesto a la luz UV, iniciando el "Proceso" en el que se produce un cambio de color debido a la exposición UV. La "Salida" muestra el resultado visible de este proceso: el cambio de color del pigmento. El ciclo de "retroalimentación" indica la naturaleza reversible de la reacción fotocrómica, que permite al pigmento volver a su estado original en ausencia de radiación UV, listo para los siguientes ciclos de detección.

El alginato es un polisacárido derivado de las algas pardas que comienza a organizarse en torno a cationes divalentes como el calcio, creando una membrana gelatinosa alrededor de un centro líquido. La interacción entre las cadenas de alginato y el calcio se ha descrito como el modelo de la "caja de huevos" (Figura S2), en el que los iones de calcio interactúan con dos cadenas de alginato, intercalándose entre ellas. Este proceso de polimerización es crucial para facilitar la formación de la membrana, ya que implica la exposición de la solución de alginato a los iones de calcio.

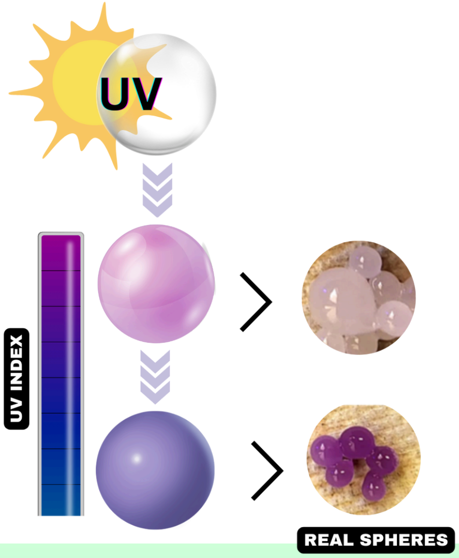

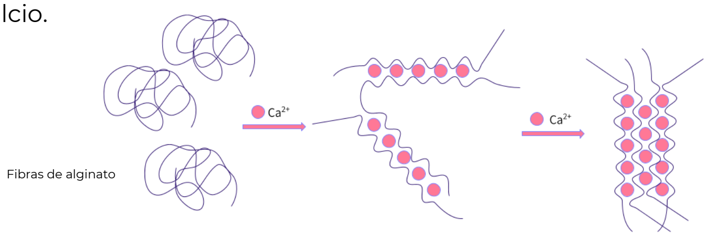

Figura S2. Modelo de caja de huevos para el proceso de esferificación del alginato. Las líneas azules representan las fibras de alginato.

Algunos de los usos y aplicaciones del alginato son: administración controlada de fármacos, ingeniería de tejidos y encapsulación de células, encapsulación de sabores en la ciencia de los alimentos, microencapsulación de microorganismos, inmovilización de enzimas y biocatálisis, órganos artificiales y terapias celulares, y biosensores, entre otros.

Estas esferas de alginato fotocrómico pueden utilizarse como herramienta educativa, ya que cambian de color con la luz ultravioleta, simulando cómo los materiales pueden indicar la presencia de radiación, de forma similar a cómo los sensores advierten de los altos niveles de radiación en el espacio. Estas esferas responden a la luz ultravioleta de igual modo que los sensores de las naves espaciales y los trajes espaciales que vigilan los distintos niveles de radiación para garantizar la seguridad de los astronautas.

Esferas de alginato que cambian de color como sensor de radiación UV: Herramienta educativa

Materiales

- **Alginato de sodio** (100% calidad alimentaria)
  - El alginato de sodio procede de algas marrones y se utiliza mucho en gastronomía molecular, por lo que es muy fácil de obtener. El alginato de sodio está clasificado como "generalmente considerado seguro".
- **Sal de calcio** (calidad alimentaria)
  - La solución de calcio puede prepararse utilizando cualquier sal de calcio soluble, como lactato de calcio, cloruro de calcio o sulfato de calcio. El lactato de calcio se utiliza habitualmente para complementar la ingesta de calcio de las personas.
- **Pigmento fotocrómico**
  - Se puede utilizar cualquier color de pigmento fotocrómico. No hace falta mucho para ver un cambio de color. Debe ser al menos 10% de pigmento fotocrómico en la mezcla de alginato es suficiente para ver un cambio de color una vez preparada la esfera.
- **Luz UV (395nm UV)**
  - Cualquier fuente de luz UV es suficiente para ver un cambio de color. Si no se dispone de una lámpara o linterna UV, la exposición directa al sol sería suficiente. Se recomienda una mini linterna LED UV de llavero.
- **Botellas de goteros o pipeta de transferencia**
  - Se utilizan goteros o pipetas de transferencia para añadir gota a gota la solución de alginato en la solución de calcio. Si no se dispone de cuentagotas o pipetas de transferencia, también se puede añadir la solución con una cuchara. Las esferas que se formen con la cuchara serán más grandes y se recomienda dejarlas más tiempo en la solución de calcio.
- **Agitador-cucharilla**
  - La cuchara-agitador se utiliza para preparar las soluciones de alginato y calcio. También retiran las esferas de la solución de calcio y el agua de enjuague. Se puede utilizar cualquier cuchara o agitador. Recomendamos utilizar una pequeña cuchara/agitador de café/té desechable para que quepa mejor en las tazas.
- **Vasos graduados para medicamentos**
  - Los vasos graduados tienen dos funciones. La primera función es medir el volumen de agua que se utilizará para preparar las soluciones o el agua de enjuague. La otra función es preparar las soluciones en ellos. Estos vasos son pequeños, fáciles de encontrar y de limpiar.
- **Papel secante**
  - Una papel secante ayudará a limpiar la zona y a colocar las esferas una vez fuera del agua de enjuague.
- **Agua**
  - Se recomiendan las botellas de agua porque son fáciles de transportar y guardar.
- **Bolsas de plástico transparente reutilizables con cierre** (1.5" x 2") (opcional)
  - Las bolsas zip sirven para almacenar las esferas y prolongar su vida útil. Las esferas de alginato se fabrican con agua y si se exponen al aire pueden deshidratarse y encogerse. Estas bolsas zip también pueden utilizarse para almacenar la mezcla de alginato (alginato + pigmento fotocrómico) o la sal de calcio (por ejemplo, el lactato de calcio) para facilitar su transporte.

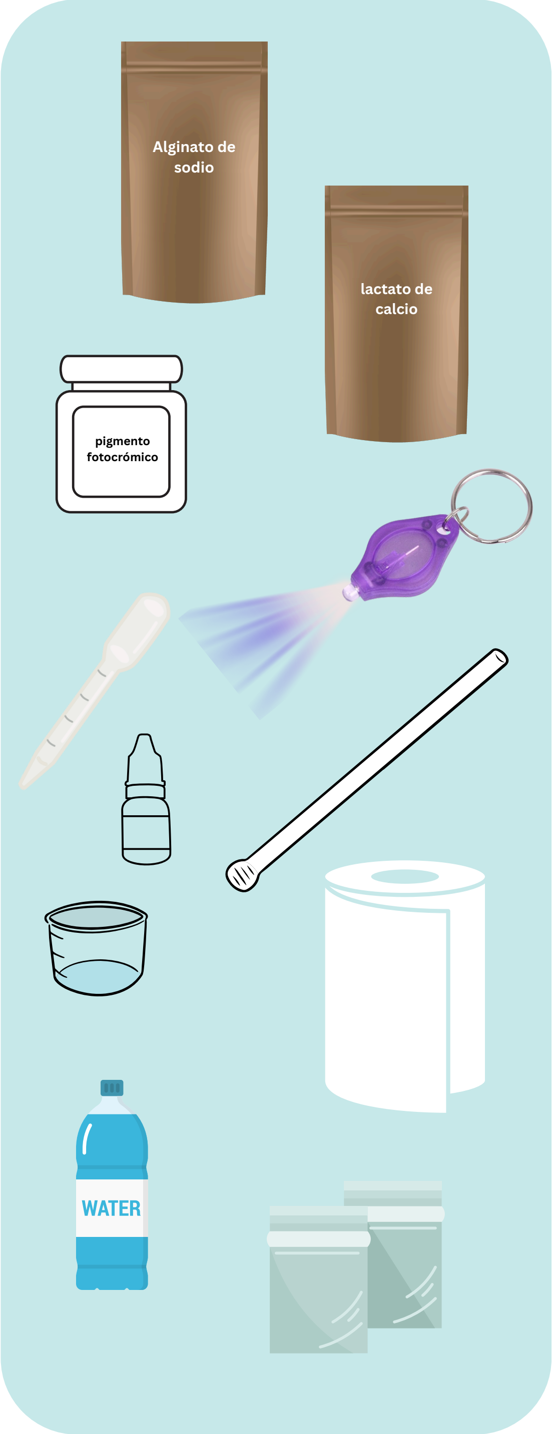

Deben tomarse las precauciones adecuadas al manipular los materiales. Elimínelos siempre de forma segura siguiendo la normativa local/nacional. No se recomienda consumir las esferas que se formen.

Esferas de alginato que cambian de color como sensor de radiación UV: Herramienta educativa

Procedimiento

Preparación

Solución de alginato

- Mezcla de alginato de sodio + pigmento fotocrómico (10%)
- 5 mL agua
- Vaso graduado de medicamento
- Agitador-cucharilla

1. Medir 5 mL de agua con uno de los vasos.
2. Añadir la punta de la espátula/agitador de la mezcla de alginato (~ 0.05 g).
3. Remover y mezclar hasta que esté bien mezclado. Utilizar el dorso de la cuchara para ayudar a disolver cualquier sólido.
4. Colocar a un lado.

Solución de calcio

- Lactato de calcio
- 10 mL agua
- Vaso graduado de medicamento
- Agitador-cucharilla

1. Medir 10 mL de agua con un vaso medidor limpio.
2. Añadir 2/3 de la espátula/agitador (~ 0.1 g) del lactato de calcio al agua y utilizar un nuevo agitador.
3. Mezclar hasta su completa disolución.
4. Colocar a un lado.

Esferificación

- Solución de calcio
- Solución alginato
- 10 - 15 mL of agua
- Vaso graduado de medicamento
- Pipeta de transferencia o agitador-cucharilla

1. Añadir gota a gota la solución de alginato a la solución de calcio. Dejar que se forme la gota antes de añadir otra gota. No introducir la punta del cuentagotas dentro de la solución.
2. Dejar las esferas en la solución durante al menos 30 segundos.
3. Enjuague las esferas. Para enjuagar las esferas, sáquelas de la solución de calcio con el agitador-cucharilla y añádales a agua fresca.
4. Deja las esferas en el agua durante al menos 30 s, después colócalas en un vaso limpio o en una toalla de papel.
5. Veamos si cambian de color al aplicar la luz ultravioleta.

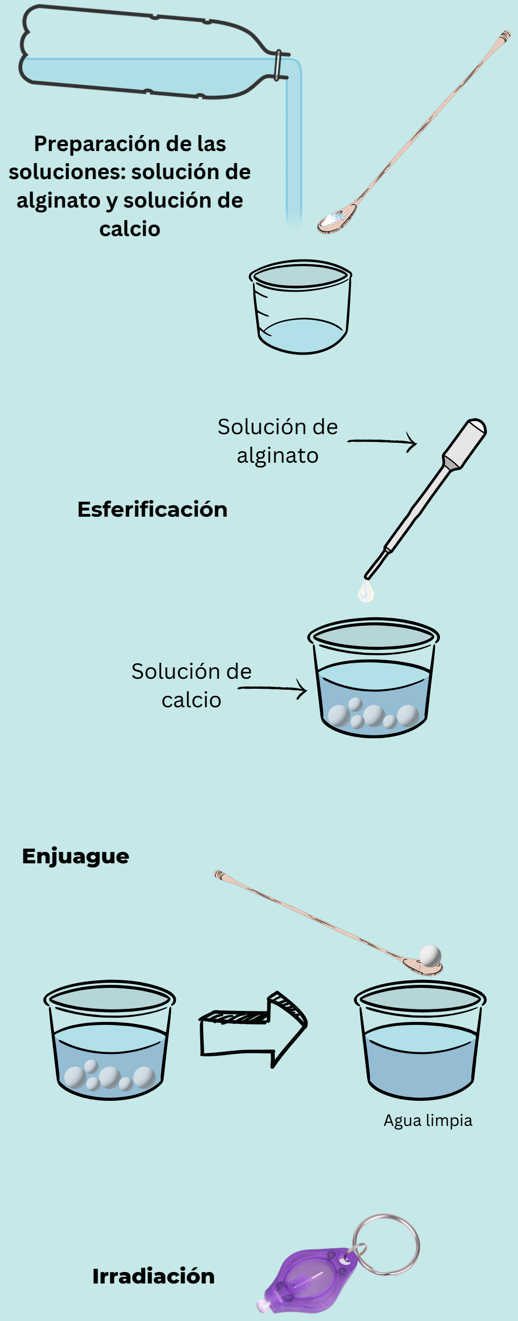

Esferas de alginato que cambian de color como sensor de radiación UV: Herramienta educativa

Información

Progressive Learning Goals for Photochromic Alginate Bead Activities Across Educational Levels

Nivel educacional	Concepto	Enfoque de la actividad	Estándar NGSS
**Elemental**	Introducción a la luz	Demostrar cómo la luz solar cambia el color de las cuentas; explicación sencilla de la luz ultravioleta.	2-PS1-1
	Química básica	Mostrar que la mezcla de alginato e iones de calcio crea esferas; discutir las propiedades observables.	2-PS1-1
**Escuela Intermedia**	Radiación UV y protección	Explicar la función y los efectos de la luz ultravioleta; introducir la protección contra los rayos ultravioleta mediante esferas.	5-PS1-3
	Modelo de partículas de la materia	Discutir la naturaleza de partícula de la materia, utilizando esferas para modelar átomos y moléculas demasiado pequeños para ser vistos.	5-PS1-1
	Reacciones químicas	Investigar cómo interactúan las sustancias durante la formación de microesferas para determinar si se ha producido una reacción química.	5-PS1-4, MS-PS1-2
**Escuela Superior**	Fotocromismo	Explorar los cambios en la estructura molecular de las esferas tras la exposición a los rayos UV; discutir las propiedades y reacciones químicas.	HS-PS1-2
	Ciencias Ambientales	Debatir las repercusiones medioambientales de la radiación UV, relacionando los materiales sintéticos con los recursos naturales.	MS-PS1-3
**Subgraduado**	Química de polímeros	Examinar la polimerización en la formación de microesferas; discutir el alginato como polímero y sus propiedades.	(Aplicaciones avanzadas)
	Espectroscopía	Analizar el cambio de color de las cuentas mediante espectroscopía; comprender las diferentes regiones del espectro de absorción.	(Aplicaciones avanzadas)
**Educación de adultos/Informal**	Comunicación científica	Involucrar al público en debates científicos utilizando esferas para explicar el cambio climático, la vigilancia de los rayos UV y la alfabetización científica.	(Participación comunitaria)

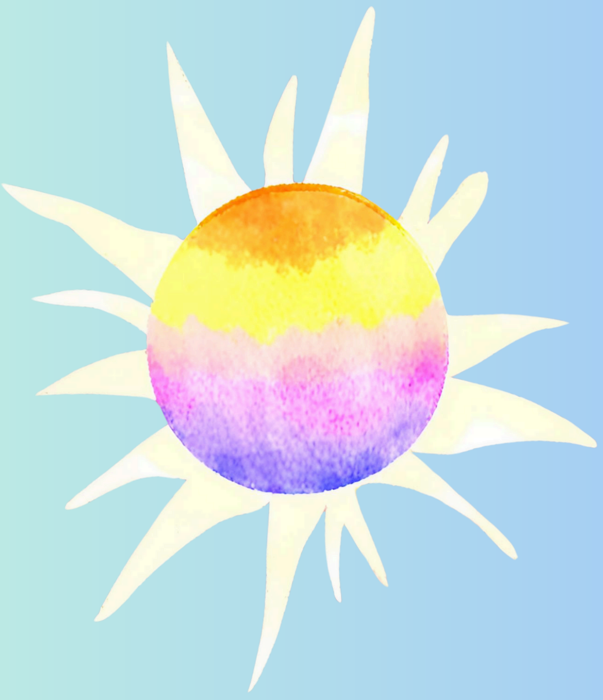

Supplement: Supplementary file 2 — ed4c00291_si_002.pdf [file ed4c00291_si_002.pdf]
