# Supplementary material for: Alginate-Based UV Sensor: A Simple and Inexpensive Tool for Educational Purposes
Source: J Chem Educ. 2024 Jul 9;101(8):3596–602. doi: 10.1021/acs.jchemed.4c00291 (PMC11328127; doi:10.1021/acs.jchemed.4c00291)
Supplement: Supplementary file 3 — ed4c00291_si_003.pdf [file ed4c00291_si_003.pdf]

Supporting Information

Alginate-based UV Sensor: A Simple and Inexpensive Tool for Educational Purposes

Kariluz Dávila-Díaz*, Liz M. Díaz-Vázquez
University of Puerto Rico, Rio Piedras Campus
17 Ave Universidad STE 1701
San Juan PR 00925-2537

*kariluz.davila@upr.edu

WHY IT MATTERS?

As the world changes and we face the depletion of the ozone layer, we face the consequence of increased exposure to the sun's rays, including ultraviolet radiation. It's crucial to understand that radiation exposure can have severe implications for human health. For instance, extended exposure to UV rays can significantly increase the risk of skin cancer and may even lead to the development of cataracts. To avoid these harmful effects, it's imperative to take the necessary precautions to protect ourselves from radiation exposure and ensure our safety.

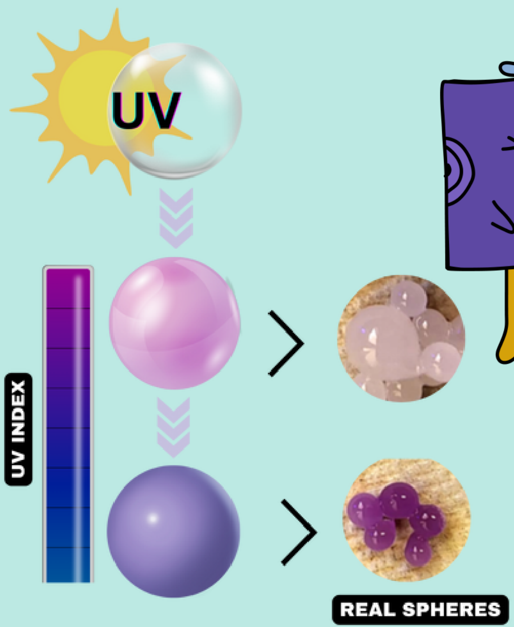

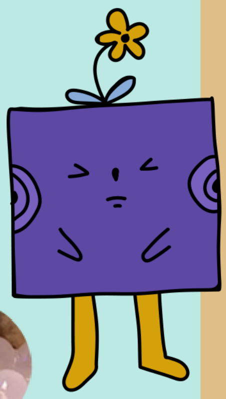

PHOTOCHROMISM

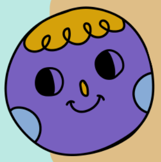

Photochromic alginate beads with safe and easy-to-find materials can be used to create sensors.

These pigments can change color and absorb light in different regions of the radiomagnetic spectrum. Photochromism is widely used in sunglasses, data storage, toys, cosmetics, clothes, supramolecular chemistry, and solar energy storage. These applications have been utilized to create sensors. Figure S1 illustrates the essential principle of using photochromic pigments in sensor production.

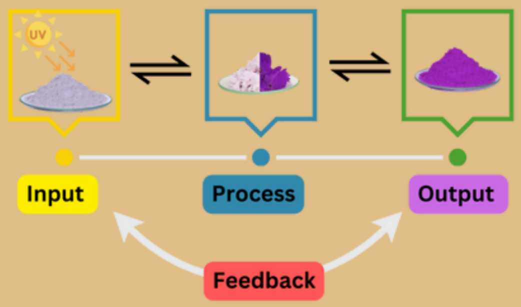

Figure S1. This is a diagram of a sensor that uses a type of pigment that changes color when exposed to UV light. When the pigment is exposed to UV light (Input), it changes color (Output), which is visible to the eye. The reaction is reversible, meaning that when the pigment is no longer exposed to UV light, it will return to its original state, ready to be used again. This process can be repeated many times to detect UV radiation.

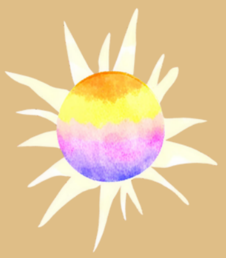

Color changing Alginate beads

SENSOR FOR UV-RADIATION

ALGINATE

Alginate is a polysaccharide derived from brown seaweeds that starts to organize around these cations in the presence of divalent cations such as calcium, creating a gel-like membrane around a liquid center. The interaction between alginate strands and calcium has been described as the "egg-box" model (Figure S2), where the calcium ions interact with two alginate strands, getting them in between. This polymerization process is crucial in facilitating the formation of a membrane, as it involves the exposure of the alginate solution to calcium ions.

Figure S2. Egg-box model for the alginate spherification process. The blue lines represent alginate strands.

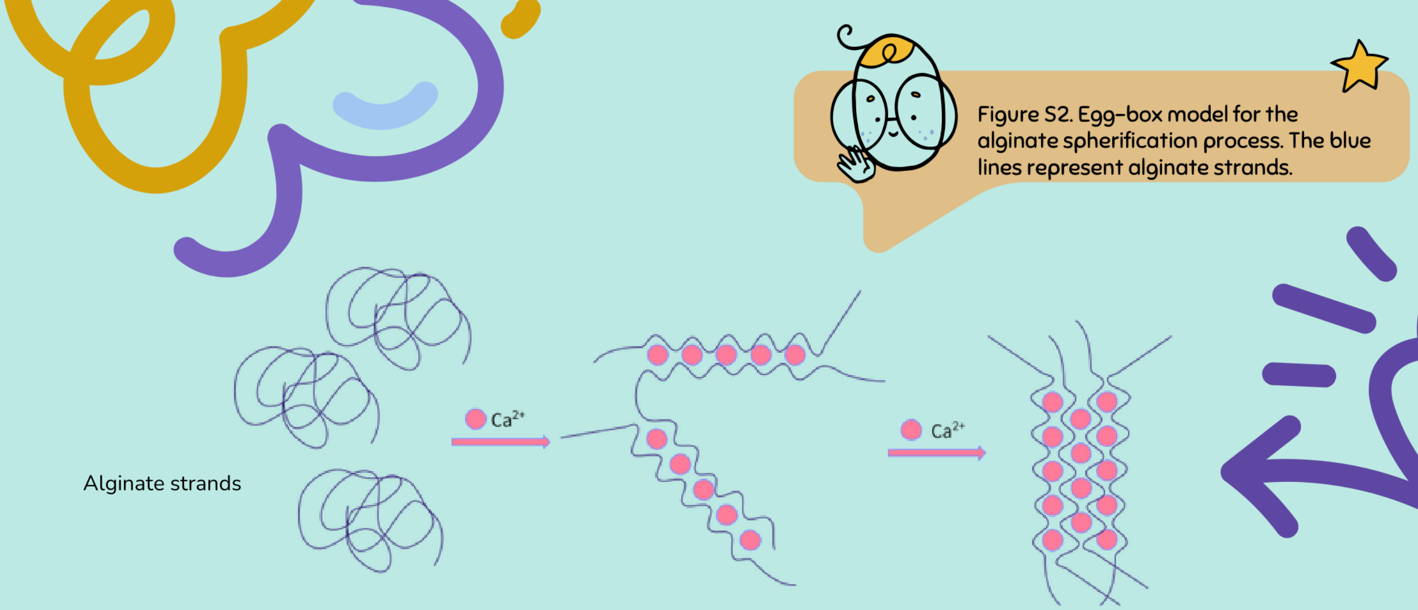

Sodium alginate is a versatile ingredient that is commonly used in various industries such as food, pharmaceuticals, and cosmetics. In the food industry, sodium alginate is often employed as a thickening and gelling agent. It is particularly popular in molecular gastronomy for creating unique textures and shapes, such as in spherification techniques.

In the pharmaceutical industry, sodium alginate is utilized to produce tablets and capsules due to its ability to bind ingredients together. It is also found in wound dressings and dental impression materials for its excellent absorption properties.

Moreover, in the cosmetics industry, sodium alginate is present in a wide range of products, including lotions, creams, and masks. Its moisturizing and soothing properties make it a popular choice for skincare formulations.

Overall, the diverse applications of sodium alginate highlight its importance as a functional ingredient across various sectors.
