# Supplementary material for: Alginate-Based UV Sensor: A Simple and Inexpensive Tool for Educational Purposes
Source: J Chem Educ. 2024 Jul 9;101(8):3596–602. doi: 10.1021/acs.jchemed.4c00291 (PMC11328127; doi:10.1021/acs.jchemed.4c00291)

Supporting Information

Alginate-based UV Sensor: A Simple and Inexpensive Tool for Educational Purposes

Kariluz Dávila-Díaz*, Liz M. Díaz-Vázquez
University of Puerto Rico, Rio Piedras Campus
17 Ave Universidad STE 1701
San Juan PR 00925-2537

*kariluz.davila@upr.edu

¿POR QUÉ ES IMPORTANTE?

A medida que el mundo cambia y nos enfrentamos al deterioro de la capa de ozono, nos enfrentamos a la consecuencia de una mayor exposición a los rayos del sol, incluida la radiación ultravioleta. Es crucial comprender que la exposición a la radiación puede tener graves consecuencias para la salud humana. Por ejemplo, una exposición prolongada a los rayos UV puede aumentar significativamente el riesgo de cáncer de piel e incluso puede provocar la aparición de cataratas. Para evitar estos efectos nocivos, es imprescindible tomar las precauciones necesarias para protegernos de la exposición a la radiación y garantizar nuestra seguridad.

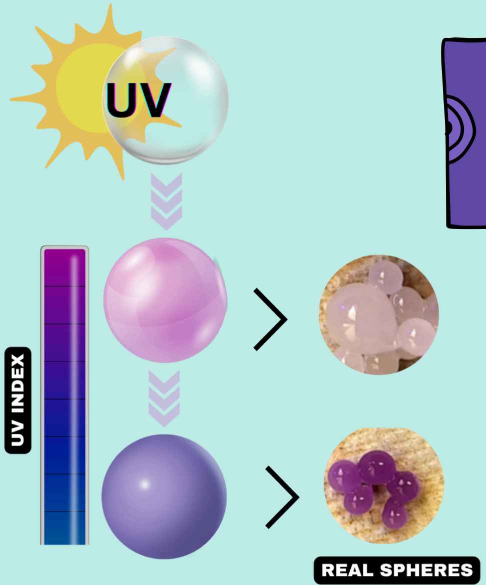

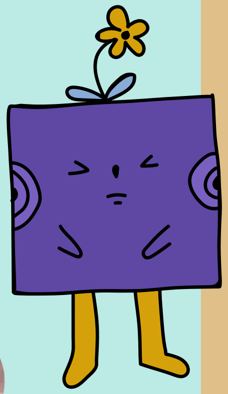

FOTOCROMISMO

Para crear sensores se pueden utilizar esferas de alginato fotocrómico con materiales seguros y fáciles de encontrar.

Estos pigmentos pueden cambiar de color y absorber la luz en distintas regiones del espectro radiomagnético. El fotocromismo se utiliza mucho en gafas de sol, almacenamiento de datos, juguetes, cosméticos, ropa, química supramolecular y almacenamiento de energía solar. Estas aplicaciones se han utilizado en la creación de sensores. La figura S1 ilustra el principio esencial de la utilización de pigmentos fotocrómicos en la producción de sensores.

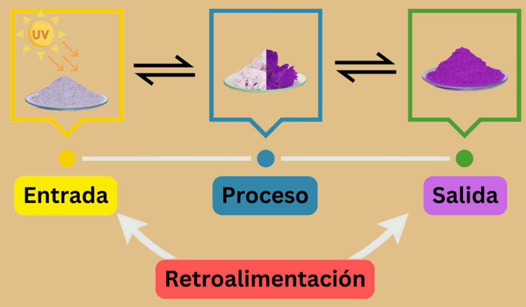

Figura S1. Este es un diagrama de un sensor que utiliza un tipo de pigmento que cambia de color cuando se expone a la luz UV. Cuando el pigmento se expone a la luz UV (entrada), cambia de color (salida), que es visible para el ojo. La reacción es reversible, lo que significa que cuando el pigmento deja de exponerse a la luz UV, vuelve a su estado original, listo para ser utilizado de nuevo. Este proceso puede repetirse muchas veces para detectar la radiación UV.

Esferas de alginato que cambian de color

SENSOR DE RADIACIÓN UV

ALGINATO

El alginato es un polisacárido derivado de las algas pardas que comienza a organizarse en torno a cationes divalentes como el calcio, creando una membrana gelatinosa alrededor de un centro líquido. La interacción entre las cadenas de alginato y el calcio se ha descrito como el modelo de la "caja de huevos" (Figura S2), en el que los iones de calcio interactúan con dos cadenas de alginato, atrapándolas entre ellas. Este proceso de polimerización es crucial para facilitar la formación de una membrana, ya que implica la exposición de la solución de alginato a los iones de calcio.

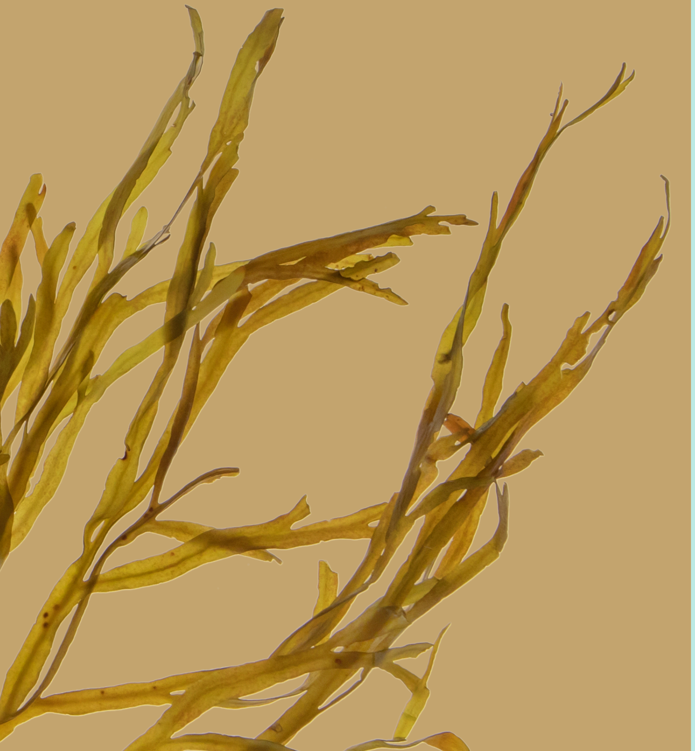

Figura S2.
Modelo de caja de huevos para el proceso de esferificación del alginato.

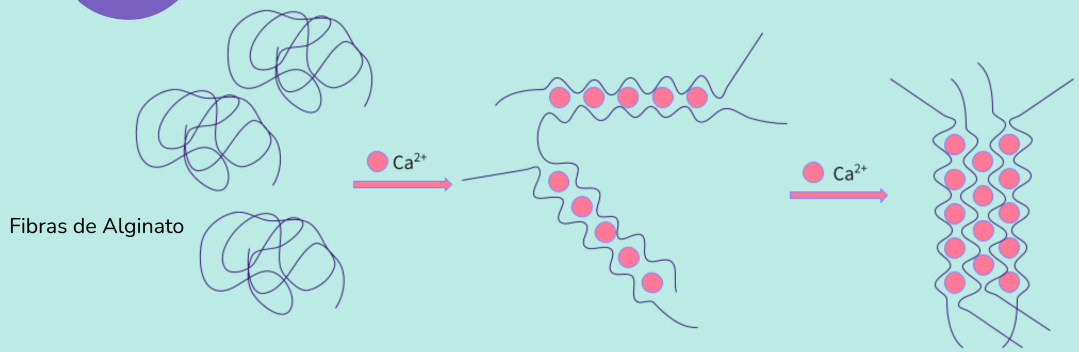

El alginato de sodio es un ingrediente versátil que se utiliza habitualmente en diversas industrias, como la alimentaria, la farmacéutica y la cosmética. En la industria alimentaria, el alginato de sodio se emplea a menudo como agente espesante y gelificante. Es especialmente popular en la gastronomía molecular para crear texturas y formas únicas, como en las técnicas de esferificación.

En la industria farmacéutica, el alginato de sodio se utiliza en la producción de tabletas y cápsulas por su capacidad para aglutinar ingredientes.
También se encuentra en vendajes y materiales de impresión dental por sus excelentes propiedades de absorción.

Además, en la industria cosmética, el alginato de sodio está presente en una amplia gama de productos, como lociones, cremas y mascarillas. Sus propiedades hidratantes y calmantes lo convierten en una elección popular para formulaciones para el cuidado de la piel.

En general, las diversas aplicaciones del alginato de sodio ponen de relieve su importancia como ingrediente funcional en diversos sectores.

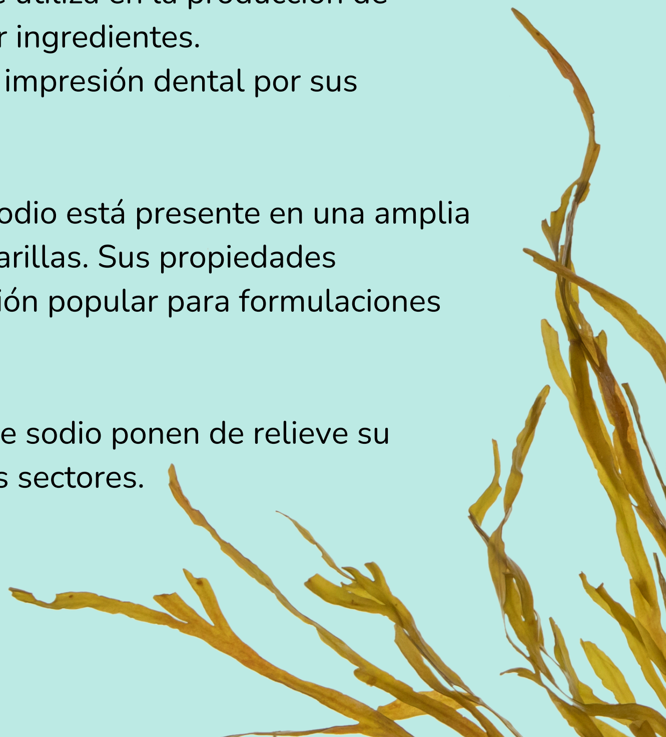

Supplement: Supplementary file 4 — ed4c00291_si_004.pdf [file ed4c00291_si_004.pdf]
